# Association between Polymorphisms in the *TSHR* Gene and Graves' Orbitopathy

**DOI:** 10.1371/journal.pone.0102653

**Published:** 2014-07-25

**Authors:** Beata Jurecka-Lubieniecka, Rafal Ploski, Dorota Kula, Konrad Szymanski, Tomasz Bednarczuk, Urszula Ambroziak, Kornelia Hasse-Lazar, Lidia Hyla-Klekot, Andrzej Tukiendorf, Zofia Kolosza, Barbara Jarzab

**Affiliations:** 1 Maria Sklodowska-Curie Memorial Cancer Center and Institute of Oncology, Gliwice Branch, Department of Nuclear Medicine and Endocrine Oncology, Gliwice, Poland; 2 Department of Medical Genetics, College of Forensic Medicine, Medical University of Warsaw, Warsaw, Poland; 3 Department of Internal Medicine and Endocrinology, Medical University of Warsaw, Warsaw, Poland; 4 Pediatric and Oncology Center, Chorzów, Poland; 5 Department of Epidemiology, Gliwice Branch, Maria Sklodowska-Curie Memorial Cancer Center and Institute of Oncology, Gliwice, Poland; Istanbul University, Turkey

## Abstract

**Background:**

Graves' orbitopathy (GO) as well as Graves' disease (GD) hyperthyroidism originate from an autoimmune reaction against the common auto-antigen, thyroid-stimulating hormone receptor (TSHR). GO phenotype is associated with environmental risk factors, mainly nicotinism, as well as genetic risk factors which initiate an immunologic reaction. In some patients GO is observed before diagnosis of GD hyperthyroidism, while it can also be observed far after diagnosis. The intensity of GO symptoms varies greatly in these patients. Thus, the pathogenesis of GD and GO may correlate with different genetic backgrounds, which has been confirmed by studies of correlations between GO and polymorphisms in cytokines involved in orbit inflammation. The aim of our analysis was to assess genetic predisposition to GO in young patients (age of diagnosis ≤30 years of age), for whom environmental effects had less time to influence outcomes than in adults.

**Methods:**

768 GD patients were included in the study. 359 of them had clinically evident orbitopathy (NOSPECS ≥2). Patients were stratified by age at diagnosis. Association analyses were performed for genes with a known influence on development of GD - TSHR, HLA-DRB1, cytotoxic T-lymphocyte antigen 4 (CTLA4) and lymphoid protein tyrosine phosphatase (PTPN22).

**Results:**

The rs179247 TSHR polymorphism was associated with GO in young patients only. In young GO-free patients, allele A was statistically more frequent and homozygous carriers had a considerable lower risk of disease incidence than patients with AG or GG genotypes. Those differences were not found in either elderly patients or the group analyzed as a whole.

**Conclusions:**

Allele A of the rs179247 polymorphism in the TSHR gene is associated with lower risk of GO in young GD patients.

## Introduction

Orbitopathy is an inflammatory autoimmune reaction involving the orbit [1]–[2]. The primary autoantigen in GO and GD is TSHR which plays a key role in triggering the onset of disease [3]. Autoimmune reactions against TSHR located on fibroblasts prompt changes in the orbit connective tissue, which create fundamental clinical signs of disease [1]–[2], [4]. Despite a common autoantigen, clinical symptoms of orbitopathy are diverse, starting from minimal symptoms to severe sight-threatening forms. Approximately one half of Graves' patients do not have clinically relevant GO [5]. GD hyperthyroidism may coexist with GO, however, most typically GO precedes or follows GD hyperthyroidism at intervals ranging from a few months to a few years [6].

Taking into account the diverse nature of the GO phenotype, one may presume the etiological factors behind GD are different from those leading to GO development. It has been proven that GO is a result of interactions between both genetic and environmental factors [7]–[9]. It has been confirmed that GO risk increases with age and is considerably higher in smokers [7], [10]–[11].

However, the role of genetic factors in the development of GO remains controversial.

Current studies suggest a correlation between polymorphisms in cytokines involved in orbit inflammation and GO, but not GD [12]–[16]. However, a majority of studies do not find differences in polymorphisms within protein-coding genes involved in immune responses or the TSHR gene, between GD patients with and without orbitopathy [9], [17]–[20].

Genetic factors may play a greater role than environmental factors in young GD patients [21]–[23]. An understanding of genetic predisposition to GO in young patients would allow the introduction of preventative measures, including smoking avoidance as well as early detection and treatment of hyperthyroidism [24]. In light of currently insufficient methods to treat GO, the potential for prevention is of particular importance [5]. Thus our study sought to analyse the genetic background of orbitopathy in different age groups, with special emphasis on genes with a known role in development of GD: TSHR, HLADRB1, CTLA4 and PTPN22.

The study revealed an association between an TSHR polymorphism and orbitopathy development in a young Polish population.

## Methods

Genomic DNA was extracted from whole blood according to standard protocols by salt protein precipitation. HLA-DRB1 typing was performed using sequence-specific oligonucleotides (SSO, Innolipa HLA-DRB1, Innogenetics, Gent, Belgium) and sequence-specific primers (MSSP Class II DRB Only, One Lambda, Dynal All Set SSP DR test, Dynal Biotech, Oslo, Norway) (Kula 2006) [25] in the Gliwice study, and by Dynal All Set SSP Dr test (Dynal Biotech, Bromborough, Wirral, UK) in the Warsaw study (Bednarczuk 2004) [26]. CTLA-4 and PTPN22 were analysed by PCF-RFLP methods, as previously described (Jurecka-Lubieniecka 2013 [27], Kula 2006 [25], Bednarczuk 2003 [28], Skórka 2005 [29]) [Table 1]. In the Gliwice study PCR was performed with 0.5 units Hot Star Taq polymerase (Qiagen). PCR products were visualized on a 2% agarose gel stained with ethidium bromide and digested with the appropriate restriction endonuclease for 3 h at 37°C. Digestion products were separated on a 3% agarose gel. Genotyping of rs179247 and rs12101255 polymorphisms in the *TSHR* gene was performed using TaqMan SNP genotyping technology (Applied Biosystems, Foster City, USA) according to the manufacturer's protocol (Ploski 2010) [30].

**Table 1:** Polymorphisms and methods used in the Gliwice study.

Gene	Polymorphism (formerly) rs (HapMap)	Location	Method	PCR Primer F 5′-3′ Primer R 5′-3′	PCR Annealing temperature	RFLP enzyme
**HLA-DRB1**	-	exon 2	PCR-SSP/PCR-SSO	-	-	-
**CTLA-4**	A(49)G rs231775/rs57563726	codon 1, exon 3	PCR-RFLP	F:CCAAGTCTCCA CTTAGTTATCC R:CCTCCATCTTC ATGCTCC	55,1°C	Bst71I, New England Biolabs
**PTPN22**	C(1858)T rs2476601	codon 620, exon 14	PCR-RFLP	F:TCACCAGCTTC CTCAACCACA R:GATAATGTTGC TTCAACGGAATTT	60°C	XcmI, New England Biolabs
**TSHR**	rs179247	intron 1	TaqMan SNP genotyping	-	-	-
**TSHR**	rs12101255	intron 1	TaqMan SNP genotyping	-	-	-

### Patients

The analysis included 768 GD patients. Patients were divided into two groups using the criterion of age of onset: younger patients being diagnosed at ≤30 years of age (n = 226), and older patients with an age of onset >30 years of age (n  =  542). Patients were consecutively recruited in the Department of Nuclear Medicine and Endocrine Oncology, Centre of Oncology in Gliwice, Poland (n = 370) and in the Department of Endocrinology, Medical University of Warsaw, Poland (n = 398). All GD patients in the cohorts investigated in the current study were unrelated and gave informed written consent. The project was approved by the relevant local research committees, including Medical Research Centre, Polish Academy of Science ethics committee and the Maria Sklodowska-Curie Memorial Cancer Centre and Institute of Oncology, Gliwice Branch ethics committee. Analysis was performed between 2003 and 2011, and median time of patient observation was approximately 5 years. All individuals were Caucasian. Criteria for the diagnosis of GD were the same for both patient groups, and were based on clinical and biochemical symptoms of hyperthyroidism, diffuse thyroid radioiodine uptake and/or detectable TSHR autoantibodies (Brahms). The severity of orbitopathy was assessed according to the NOSPECS classification. The clinical characteristics of GD patients are presented in Table 2.

**Table 2:** Clinical characteristics of patients with GD, N = 768.

Gender (female: male)	617∶151
Age at diagnosis of GD in years (mean ± SD)	40.3±14.49
GO present (NOSPECs≥2)	359 (46.7%)
Tobacco smokers	322 (41.9%)
Disease duration in years: (mean ± SD)	2.72±4.38

### Statistical analysis

Statistical analysis was performed using the statistical program STATA12.0. To ensure genotyping accuracy, Hardy-Weinberg equilibrium was calculated. The distribution of genotypes and alleles were compared between groups using the Chi-square test and Fisher's exact test [where appropriate]. All tests were two-sided, and p<0.05 was considered significant. Odds ratios [ORs] were calculated with 95% confidence intervals (95% CI) to measure the effect of study factors on risk of GO. Association analyses of polymorphisms were performed for age of diagnosis (≤30 years vs. >30 years), severity of orbitopathy based on NOSPECS classification (NOSPECS 0–1 vs. NOSPECS 2–6), and smoking status. A multivariate logistic regression was used to determine the independent association between GO and the above factors.

To establish types of patients, a cluster analysis was applied. This analysis formed subsets of patients (clusters) by minimizing within-group variance and maximizing between-group variance using the chosen measure of dis/similarity. The following risk factors were taken into account: smoking, *TSHR* rs179247 polymorphism, and age at GO onset. Once the patient groups were structured in clusters, they were able to be interpreted to explain the membership of patients in the clusters and their underlying predicting factors. This method may provide an alternative explorative platform for the identification of such predictors. Taxonomy uses a wide range of algorithms to determine the distance between objects. In our study, we used the Marczewski-Steinhaus algorithm, which relies on the use of a symmetric difference between objects [31].

## Results

### Allele frequency and genotype distribution analysis

The genotype frequency in the GD group was in Hardy-Weinberg equilibrium.

In order to assess genetic predisposition to GO, we compared patterns of polymorphisms in the TSHR, PTPN, CTLA4 and HLA DRB1 genes in patients with and without orbitopathy. Results were analysed both for the group as a whole group, as well as for subgroups of younger (age at diagnosis 30 or less) and older (age at diagnosis greater than 30) patients.

**Figure 1:**
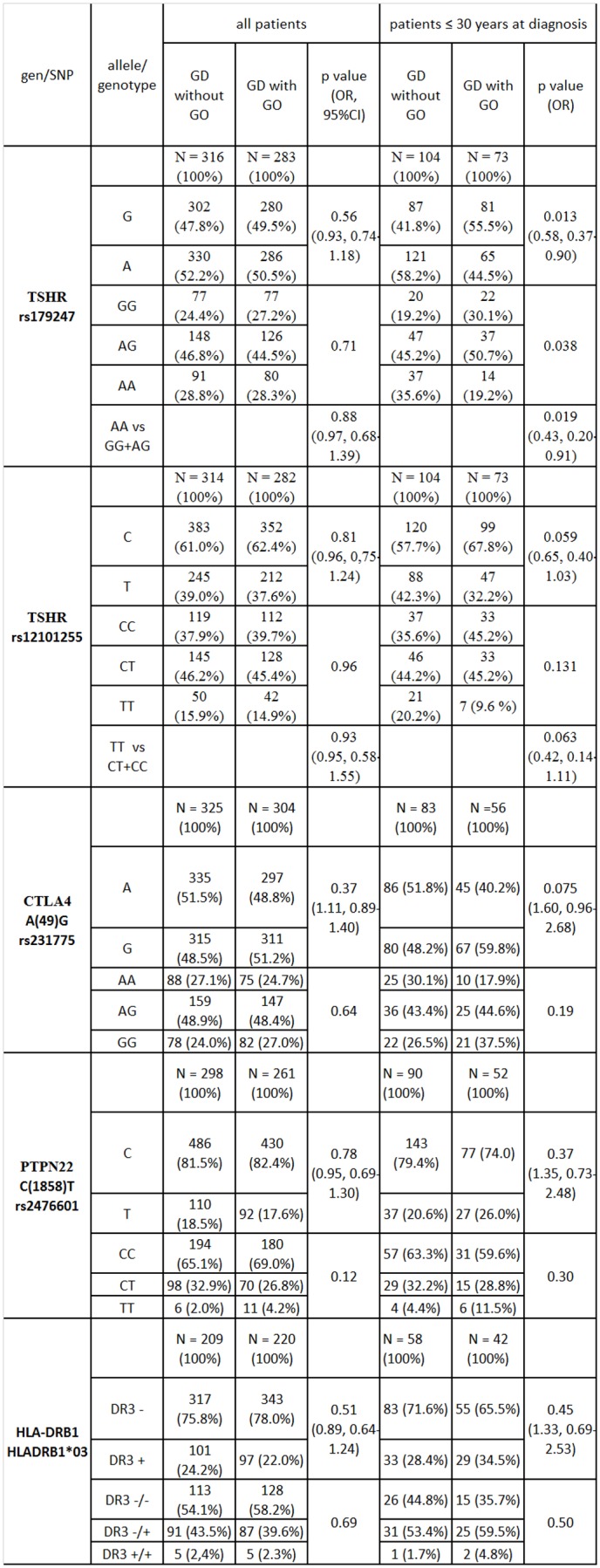
Frequencies of alleles and genotypes in Graves' patients with and without orbitopathy.

When the group was analyzed as a whole, as well as when older patients were analyzed alone, there was no difference found in allele frequency or distribution of genotypes for any of the analyzed polymorphisms. Analysis of the younger patient group revealed significant differences in the presence of polymorphisms in relation to development of GO. Results of allele and genotype frequency analyses are presented in Figure 1.

#### TSH receptor

Two polymorphisms in the TSHR gene (rs179247 and rs12101255) with a known association to GD in Polish populations were analyzed. It has been proven that allele A of rs179247 and allele T of rs12101255 are more frequent in GD patients. Allele G of rs179247 and allele C of rs12101255 are more common in the healthy population [30]. In our study, in the group of younger patients, allele A of polymorphism rs179247 was found significantly more frequently in patients without GO in comparison to those with GO (58.2% vs. 44.5%, p = 0,013, OR = 0.58) (Figure 1). In this group there was a statistically significant difference in genotype distribution (p = 0.038). The presence of a homozygous AA was associated with a significant reduction in risk of disease incidence, as compared to patients with AG or GG genotypes (p = 0.019, OR = 0.43) (Figure 1). Analysis of the rs12101255 polymorphism in younger patients showed an increased presence of allele T and homozygous TT genotypes in patients without orbitopathy. This increase in frequency fell short of statistical significance (42.3% vs. 32.2% p = 0.059 and 20.2% vs. 9.6%, p = 0.063 respectively). Of the 599 patients with GD, in which the TSHR polymorphisms were determined, analysis of 177 patients young with and without GO showed a relationship between polymorphism rs179247 of TSHR gene and the occurrence of GO (p = 0.013) with the power of 76%.

#### CTLA4

Allele G was found more frequently in younger patients with GO in comparison to those without GO. However, this observation was not statistically significant (59.8% vs. 48.2%; p = 0.075; OR = 1.60) (Figure 1)

#### PTPN22

Arrangement of alleles and genotypes of PTPN22 C(1858)T/ rs2476601 did not differ in patients with and without GO (Figure 1).

#### HLA DRB1*03

Arrangement of alleles and genotypes of polymorphism HLA DRB1*03 did not differ in patients with and without GO (Figure 1).

### Haplotype frequency analysis

The analyzed polymorphisms, rs12101255 and rs179247 show linkage disequilibrium (LD). D'is very close to 1.0 (D' = 0.97), which indicates a strong linkage disequilibrium at p  = 2.2204e-16. The most common haplotype in patients with GD is the haplotype comprising two AT-risk alleles. It occurs more frequently in patients with GD than in healthy subjects (37.6% vs. 26.2%, OR  = 1.72, p = 0.00002) (data unpublished).

### Logistic regression

Multivariate logistic regression was performed using GO as the dependent variable and the studied polymorphisms, age of onset and smoking status as the independent variables.

Results of the logistic regression confirmed observations from analyses of alleles and genotypes. Genetic predisposition and smoking are independent risk factors with influences on development of orbitopathy in younger patients. The presence of the rs179247 polymorphism in the TSH receptor gene significantly lowers the risk of GO incidence (OR = 0.571, p = 0.012). However, in older patients smoking is the only significant factor influencing development of orbitopathy (OR = 1.802, p = 0.001) (Table 3).

**Table 3:** Results of multiple linear regression analysis.

	OR	95% confidence interval	p-value
**Age at diagnosis ≤30** TSHR rs179247 Smoking	0.571	0.37–0.88	0.012
	1.879	0.97–3.66	0.063
**Age at diagnosis >30** Smoking	1.802	1.27–2.56	0.001

### Taxonomy

**Figure 2:**
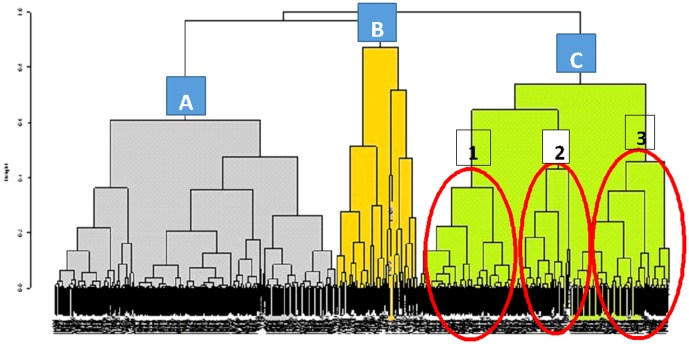
Dendrogram: age at diagnosis, smoking, TSHR rs179247. Group A: N = 249, median age 43 years, smokers, genotypes: GG, AG, AA, with GO: N = 138 (55%), without GO: N = 112 (45%). Group B: N = 73, median age 41 years, non smokers, genotypes: GG with GO: N = 33 (45%), without GO: N = 41 (55%). Group C: N = 235, median age 35 years, non smokers, genotypes: AA,AG. with GO: N = 90 (38%), without GO: N = 145 (62%). Subgroup C1: N = 85, median age: 48 years, genotypes: AG with GO: N = 37 (44%), without GO: N = 48 (56%). Subgroup C2: N = 59, median age: 22 years, genotypes: AG With GO: N = 23 (39%), without GO: N = 36 (61%). Subgroup C3: N = 91, median age 35 years, genotypes: AA With GO: N = 30 (33%), without GO: N = 61 (67%).

In order to confirm our results, additional analyses of GO incidence among GD patients were performed and compared with results of the GO risk factor analysis (rs179247 polymorphism, smoking and age at diagnosis) obtained using logistic regression. Taxonomy was used to group patients in terms of similar values for chosen parameters. The group was divided into three subgroups (Figure 2):

Subgroup A included older smoking patients (median age 43), among which all rs179247 genotypes (AA, AG, GG) were found. Polymorphism frequency was not associated with the presence or absence of GO.

Subgroup B was comprised of non-smoking patients with a GG genotype.

**Figure 3:**
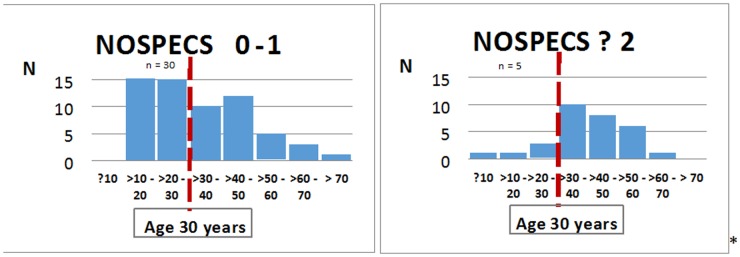
Incidence of GO in subgroup C3 with AA genotype TSHR rs 179247.

The most interesting to us was subgroup C, which included allele A carriers, non-smokers and had the lowest median age (35 years). In this subgroup a significant discrepancy in genotype distribution (AG and AA) was revealed depending on age of diagnosis and orbitopathy occurrence. Allele A carriers were dominant in the group of patients without orbitopathy (N = 145 vs. N = 90) (data unpublished). The difference was more significant in young (age of diagnosis ≤30 years of age) allele A carriers (N = 64 vs. N = 28) (data unpublished). Whereas orbitopathy was not present in 86% of young AA homozygote carriers (subgroup C3) (N = 30 vs. N = 5) (Figure 3).

## Discussion

The TSHR gene encodes the thyroid-stimulating hormone receptor (TSHR) - a protein that plays a role in the pathomechanism of GO. Thus, polymorphisms in this gene may result in a predisposition for GO [1]–[3], [9]. TSH receptor is the dominant autoantigen involved in the development of an immune response carried out by immunocompetent cells expressing HLA class II complex on the surface [32].While the cascade of immunological response is generally well known, the mechanisms leading to the origin of the MHC II-TSHR complex need to be analyzed more extensively [1], [4], [9]. The etiology of disease may result from genetic differences in the TSHR gene, leading to TSHR protein structural differences [33]–[34]. Intronic polymorphisms – not exonic – are responsible for generation of different receptors forms [34].

The TSHR gene association with GD was well established. Two independent studies identified strong association within *TSHR* intron 7 in Japanese and *TSHR* intron 1 in UK Caucasian cohorts, which provided the first convincing evidence for *TSHR* association with GD [35]–[36]. Recent detailed association mapping of 98 SNPs across 800 Kb of the *TSHR* region, refined association to within 40 Kb of *TSHR* intron 1 in 768 GD patients and 768 controls of UK Caucasian origin. Strongest SNP associations were rs179247 (OR = 1.53) and rs12101255 (OR = 1.55), which were replicated in Polish-Caucasian cohorts. A different study showed that two SNPs (rs12101261, rs179243) in intron 1 are associated with GD in Chinese population [37]. However these polymorphisms are in linkage disequilibrium with SNP's previously reported in Caucasian populations. These results strongly suggest that SNP's in intron 1 of TSHR are risk factors for GD. The role of SNP's in intron 7 remain to be established.

In our work we analyzed the association of SNPs rs12101255 and rs179247 with orbitopathy as their relationship with the development of GD was proved in the Polish population [30].

The location of rs179247 and rs12101255 within the first intron, close to the promoter region and start codon, may influence gene expression or posttranslational processes. Structurally and functionally altered TSHR isoforms become an immunotarget in patients with a genetic predisposition to GD. Polymorphisms rs179247 and rs12101255 were associated with reduced expression of full length TSHR mRNA relative to two truncated splice variants in thyroid tissue [2], [4], [33]–[34].

Our analysis demonstrated an association between orbitopathy and the TSHR gene rs179247 polymorphism. In younger patients without orbitopathy, in whom GD was diagnosed before 30 years of age, the A allele occurred significantly more often. Presence of an AA homozygous locus was associated with a significant reduction in risk of GO incidence. Independent taxonomy also confirmed the relation between TSHR polymorphism rs179247 and GO in younger patients. Orbitopathy was not present in 86% of young AA carriers (N = 30 vs. N = 5) (Figure 3). Patients with allele T or homozygous TT at the rs12101255 polymorphism in TSHR demonstrated an increased frequency in GO incidence within this group of patients (Figure 1). The AT haplotype occurs most often in patients with GD, and is associated with an increased risk of disease (OR = 1.72, p = 0.0002).

However, when all patients were analysed, or when only older patients (age at diagnosis >30) were analysed, the frequency of alleles present and genotype distributions of both polymorphisms did not differ in patients with or without GO.

These findings showed an association between the presence of TSHR polymorphisms in younger patients and the incidence of orbitopathy. Allele A of rs179247 was more frequent in GD patients regardless of age than in healthy persons [30]. In our study this allele showed a correlation with lesser risk of GO in young GD patients.

This was also confirmed by preliminary epidemiologic studies showing that GO incidence appears in two peaks throughout life – after 40 years of age and after 60 years. In these patients environmental factors (mainly nicotinism) were the factors most closely correlated with illness occurrence [10], [24]. In younger patients GD-associated eye symptoms were mild and characterized by a self-limiting course without involvement of the oculomotor nerve [38]–[41]. Current publications have not revealed a correlation between TSHR polymorphisms and orbitopathy [19]–[20]. These studies have focused on an rs2268458 polymorphism localized within the first intron of the TSHR gene. The development of GO showed no association with the TSHR polymorphism, however the patient group was relatively small and the median age was 48 [19]. In a second study, extended to 199 patients, the population was dominated by patients with severe orbitopathy and orbital decompression, with a median age of 52. There were no differences revealed in the frequency of allele C amongst GD patients with or without GO [20]. Our analysis of older patients (greater than 30 years old at diagnosis) as well as the group analyzed as a whole (median age 40) yielded similar results. Taxonomic classification allowed the separation of a group of older smoking patients (group A, median age 45), in which there was no association between the rs179247 TSHR polymorphism and orbitopathy (Figure 2).

Syed et al. analyzed the influence of the rs2268458 TSHR polymorphism, as well as seven PTPN12 polymorphisms, on the phenotypes of GD. Association of the TSHR rs2268458 polymorphism with mild/moderate orbitopathy was confirmed, however in interaction with PTPN12 polymorphisms only. Authors showed independent correlations of three PTPN12 polymorphisms with orbitopathy and interaction of each with the rs2268458 TSHR polymorphism. No age related dependencies were found [42].

The role of other polymorphisms in development of orbitopathy remains unclear.

The first studies looking at genetic background in the context of orbitopathy indicated an association between the A(49)G polymorphism in the CTLA4 gene and an increased risk of GO [43]. These conclusions were confirmed in a later analysis of the same group [44]. Other studies of Caucasian groups did not confirm the relationship between the A(49)G polymorphism and GO [10], [45]–[47].

The HLADRB1*03 polymorphism occurs most commonly in GD patients of Caucasian origin. Some papers have confirmed an association between HLADRB1*03 and GO [48]–[49]. However, other authors have not found differences in the distribution of the HLADRB1*03 allele in GD patients with or without GO [10], [27], [50]–[51]. There is no sufficient evidence to support a correlation between the C(1858)T polymorphism in the PTPN22 gene and GO [29], [42].

Our analysis of A(49)G polymorphisms in the CTLA4 gene and C(1858)T polymorphisms in the PTPN and HLADRB1*03 genes did not reveal any statistically significant discrepancies in allele frequencies or distribution in patients with or without orbitopathy in all age groups. However, there was an observed tendency showing a more frequent occurrence of allele T of the C(1858)T polymorphism in the PTPN gene, the HLADRB1*03 allele and the G allele of the A(49)G polymorphism in the CTLA4 gene in young patients with GO. One limitation of this study was the inclusion of a relatively small group of patients in particular subgroups, which causes statistical difficulties and may indicate caution in the interpretation of negative results. Future studies will include a larger group of patients.

Difficulties in the assessment of genetic predisposition to GO are in large part due to genotype heterogeneity in autoimmunological disorders. Interactions between many genes with modest increases in relative risk influence the development of particular GD phenotypes, including orbitopathy [52]. Thus it is very important to precisely classify GO patients according to the clinical course of disease [18]. Analysis of genetic predisposition is also hampered by the necessity for long-term observation, due to undefined time frames for development of hyperthyroidism and GO, as well as the confounding effects of environmental factors [6], [53].

Introduction of the described age-grouping method may allow a simplification in assessment of genetic predisposition to GO because age grouping decreases the burden of long-term observation. In the young group of patients environmental influences have had less time to play a role in disease course, therefore these environmental effects are relatively minimized. Lesser influence of the environment is an important factor to be considered in favor of the validity of a method dividing analyzed groups into age differentiated subtypes.

To continue our study we have planned a wider assessment of genetic predisposition to GO in Polish children and youth.

In summary, we have found that allele A of the rs179247 polymorphism in the *TSHR* gene is associated with lower risk of GO in young patients with GD.
